# Efficacy and safety of a new resilient hyaluronic acid dermal filler, in the correction of moderate‐to‐severe nasolabial folds: A 64‐week, prospective, multicenter, controlled, randomized, double‐blind and within‐subject study

**DOI:** 10.1111/jocd.13100

**Published:** 2019-08-24

**Authors:** Joely Kaufman‐Janette, Susan C. Taylor, Sue Ellen Cox, Susan H. Weinkle, Stacy Smith, Brian M. Kinney

**Affiliations:** ^1^ Skin Research Institute Coral Gables FL USA; ^2^ Perelman School of Medicine University of Pennsylvania Philadelphia PA USA; ^3^ Aesthetic Solutions P.A. Chapel Hill NC USA; ^4^ Bay Area Medical Complex Bradenton FL USA; ^5^ California Dermatology & Clinical Research Institute Encinitas CA USA; ^6^ Keck School of Medicine University of Southern California Los Angeles CA USA

**Keywords:** clinical trial, facial dynamism, Hyaluronic acid, nasolabial fold, resilient

## Abstract

**Background:**

Injectables that behave similarly to native tissue and preserve facial expressiveness represent a new frontier in aesthetic medicine. A range of fillers made of high molecular weight hyaluronic acid (HA) chains with low crosslinking have been specifically developed to complement facial dynamics.

**Aims:**

The efficacy and safety of one of these resilient HA fillers, and its noninferiority to an effective comparator available in the US, were tested in the treatment of dynamic wrinkles.

**Methods:**

A 15‐month, prospective, multicenter, controlled, randomized, double‐blind, within‐subject (split‐face) clinical trial was conducted on 140 subjects with moderate‐to‐severe nasolabial folds (NLF). Study endpoints included improvement on a proprietary Wrinkle Severity Rating Scale (WSRS) and Global Aesthetic Improvement Scale, according to Blind Live Evaluators (BLE), subjects, and treating investigators (TI). Subject perception was evaluated with FACE‐Q and satisfaction scales.

**Results:**

The per‐protocol population included 88 subjects (92% women) of all Fitzpatrick phototypes, with a mean age of 57 years. WSRS improvement was significantly greater with the resilient HA than its comparator over 15 months, including at week 24 (primary endpoint), as rated by BLE and TI. Results demonstrated the noninferiority of the resilient HA filler to its comparator. Aesthetic improvement and subject satisfaction were durably high, with an overall trend toward higher scores for the resilient HA filler. Both treatments were safe and well tolerated.

**Conclusion:**

The resilient HA filler made of long chains lightly crosslinked is at least equivalent to a well‐established comparator for the correction of NLF in subjects of diverse skin phototypes.

## 1 INTRODUCTION

In the last two decades, hyaluronic acid (HA)‐based fillers have become the material of choice for use in soft tissue and dermal correction.^1^, ^2^ The enduring popularity of HA fillers stems from their ability to produce immediate, predictable, and natural‐appearing results when injected appropriately, with excellent safety and tolerability profiles.^3^

The portfolio of fillers available for soft tissue augmentation has expanded rapidly over the last decade, and nowadays, clinicians may choose from a vast repertoire of HA‐based fillers with different features depending on factors such as HA molecular weight, concentration, crosslinking, also known as degree of modification (MoD). Crosslinking is required for any HA filler to reduce early degradation and ensure durability; noncrosslinked HA is rapidly degraded and resorbed.^2^, ^4^, ^5^, ^6^, ^7^ Clinical applications for each filler rely on a thorough understanding of age‐related volume loss and the recognition of wrinkle physiology as a multilevel process. Injectors must also be familiar with the different rheological properties of each filler and—most importantly—how they behave in vivo when injected in different areas. Indeed, to achieve natural‐looking aesthetic results, while the depth and the area of injection are key, the rheological properties of HA fillers are also crucial, especially for injectors aiming to preserve facial expressiveness. The injected products must be able to adapt to facial movements in a similar way to native tissue.^8^ It is important to note that despite significant progress in HA filler design and manufacturing, there is still limited evidence on the clinical efficacy of many products, and comparative studies between fillers are often lacking. As a consequence, clinicians often have to make decisions based solely on information provided by manufacturers.

RHA^®^ 4 (RHA4, available outside the United States as TEOSYAL RHA^®^ 4) is one of the 4 dermal fillers of the RHA (Resilient Hyaluronic Acid) range manufactured by TEOXANE SA, Geneva, Switzerland. It is made of sterile, biodegradable, viscoelastic HA crosslinked using a new technology intended to minimize the degradation of HA chains during the manufacturing process and reduce the MoD of HA in the final product, with the objective of improving adaptability to skin movements.^9^, ^10^ These optimized production conditions are designed to preserve natural HA polymers from crosslinking‐associated cleavage. The resulting high molecular weight chains form a network of entangled HA fibers which require fewer 1,4‐butanediol diglycidyl ether (BDDE)‐covalent bonds for stabilization. As a result, the MoD of RHA fillers remains low (2%‐4%) compared to most monophasic gels (5%‐10%),^7^ allowing the less rigidly crosslinked HA chains^10^ to interact and slide dynamically while maintaining in vivo durability.

The mechanical characterization of RHA gels^11^ indicates that these fillers show a resilient behavior (data on file, from TEOXANE Laboratories), *that is* improved capacities to recover their initial position and retain their mechanical features after being compressed, stretched, or bent. These features are thought to be key for HA implants to respect and accompany facial dynamics. An excellent tissue integration of the fillers has previously been reported by RHA injectors.^10^

RHA4 has been developed with mechanical properties adapted for the correction of deep wrinkles and volume loss in extended areas. Together with RHA^®^2 and RHA^®^3, these fillers were recently approved in the US by the FDA for the correction of moderate‐to‐severe dynamic facial wrinkles and folds such as nasolabial folds (NLF).

The objective of this 15‐month, controlled, randomized, double‐blind, split‐face clinical trial was to compare the efficacy and safety of RHA4 vs an effective standard of care approved in the US for the correction of NLF, Restylane^®^ Lyft with lidocaine (Lyft), manufactured by Galderma SA, Lausanne, Switzerland, and formerly known as Restylane *Perlane*‐L^®^.

## 2 MATERIALS AND METHODS

### 2.1 Trial design and population

**Figure 1:**
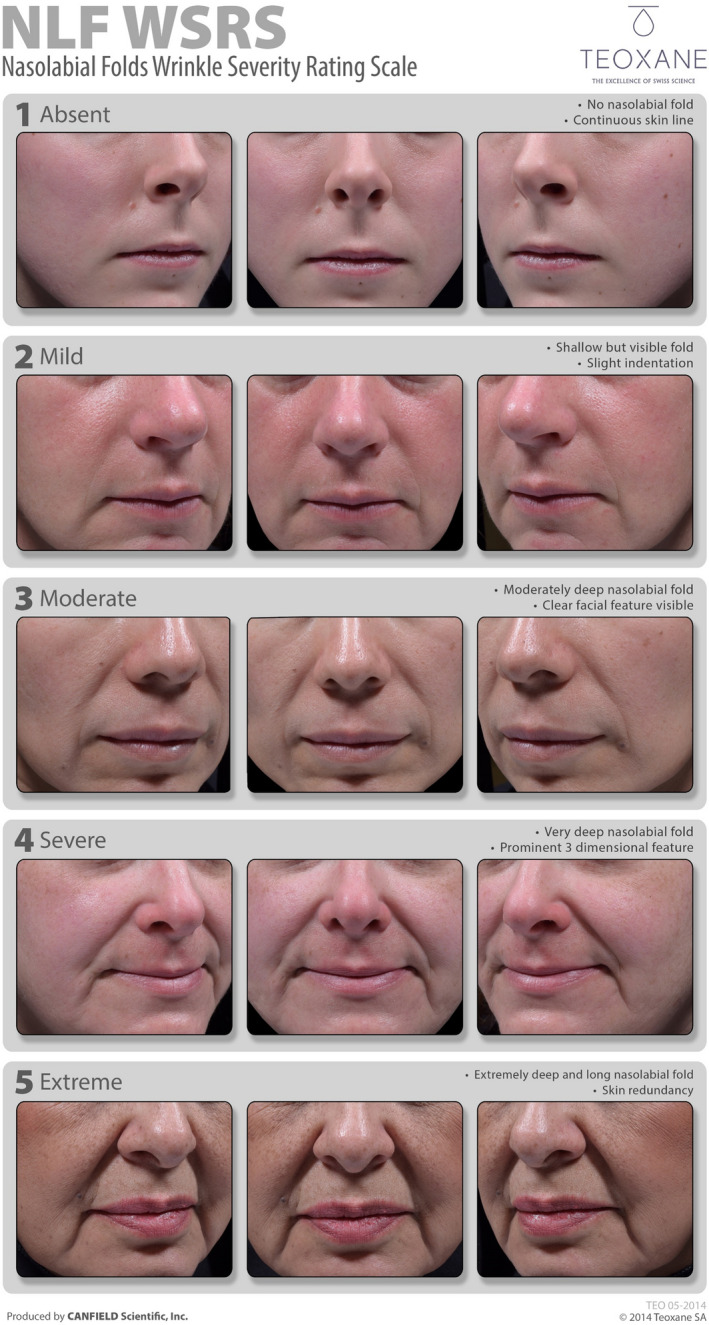
TEOXANE NLF‐WSRS 5‐point grading scale

This was a prospective, multicenter, controlled, randomized, double‐blind, within‐subject (split‐face) clinical trial to assess the noninferiority of RHA4 vs Lyft for the correction of moderate‐to‐severe NLF (ClinicalTrials.gov Identifier: NCT02253147). The study included subjects aged ≥22 years, with moderate‐to‐severe symmetrical NLF graded 3 or 4 on a proprietary and validated five‐point scale (TEO 05‐2014) developed by TEOXANE Laboratories for scoring the severity of NLF: the NLF‐Wrinkle Severity Rating Scale (NLF‐WSRS), which will be referred to as WSRS in this manuscript. Each grade on the scale represents a clinically meaningful change in NLF severity. Grade 1 indicates no visible NLF (*ie*, continuous skin line) and grade 5 indicates extreme deep and long NLF with skin redundancy (Figure 1). This scale was validated in June 2014, by measured inter‐ and intra‐repeatability of trained evaluators, following a process similar to the WSRS published in 2004.^12^

Subjects were randomized according to a 6:1 ratio to (a) the intervention group, treated with RHA4 and Lyft (one hemiface each) and (b) the untreated control group. The randomization algorithm also provided balancing for the side (*ie,* left or right) and order of injections. Subjects with known hypersensitivity or previous allergic reaction to any component of the study devices or to local anesthetics of the amide type were excluded. Main exclusion criteria also included known susceptibility to keloid formation, hypertrophic scarring or clinically significant skin pigmentation disorders, and history of connective tissue disease. Subjects also needed to be free from any permanent or semi‐permanent filler, resorbable facial filler within a year. For 6 months prior to enrollment and for the duration of the study, subjects were to refrain from any other facial aesthetic procedures such as laser, ultrasound, surgery, or peelings.

Informed consent was obtained before starting any study‐related procedures. The study was conducted in five centers in the United States between September 22, 2014 and May 12, 2016, and according to the Helsinki Declaration (1964) and its successive updates, and seeking to comply with Good Clinical Practices ICH guideline.

### 2.2 Treatment

Active treatments consisted of injections of RHA4 or Lyft, which are both crosslinked HA in concentrations of 23 mg/mL and 20 mg/mL, respectively. Both are mixed with 0.3% w/w lidocaine in a physiological buffer and supplied with 27G½” needles. Additional anesthesia was prohibited in this study. RHA4 and Lyft devices were both administered using various common injection techniques, including linear threading, multiple serial punctures, and/or fan‐like injections. Both the injection technique and depth (*ie*, deep‐dermis or superficial subcutaneous) were at the discretion of the treating investigator and were the same on both hemifaces. The treating investigator was aware of the identity of the injected product, but patients and a Blinded Live Evaluator (BLE) were blinded to the administered treatment.

Treatments were applied in two visits. At the initial treatment visit (visit 1), each subject received injections of RHA4 into the left or right NLF, and injections of Lyft into the contralateral NLF. Subjects randomized to the untreated control arm of the study did not receive treatment. Volumes injected were at the discretion of the injector to obtain optimal correction, up to a maximum of 3.0 mL/session/side, regardless of the device. Two weeks after the initial treatment, if the investigator deemed that the NLF did not have optimal correction, then a touch‐up was performed using the same product that was initially injected. Subject follow‐up was carried out for 64 weeks after the initial and any needed touch‐up injection sessions. After optimal correction, subsequent retreatments with RHA4 only were also carried out in the following situations: (a) Optional retreatment was offered at week 24 or 36, if both NLF had returned back to baseline WSRS grade, or if a ≥2 WSRS‐grade asymmetry was observed, (b) conditional retreatment was offered at week 52, if at least one NLF went back to baseline WSRS grade, or if a ≥2 WSRS‐grade asymmetry was observed. Retreated subjects then exited the study. (c) Unconditional retreatment was offered at week 64 to all subjects provided they did not receive conditional retreatment at week 52. Safety follow‐up evaluations after all retreatments were performed.

### 2.3 Study endpoints and variables

The primary endpoint was based on WSRS score improvement from baseline, as assessed by the BLE at week 24. The mean within‐subject difference, between improvement of the NLF treated with RHA4 vs the one treated with Lyft, was analyzed. WSRS grading was performed independently by the BLE, as well as treating investigators, to avoid biases. A WSRS change of ≥1 grade was considered clinically significant.

Secondary endpoints included the following: (a) WSRS score improvement (as rated by the BLE at weeks 24, 36, 52, and 64, and by the treating investigators at weeks 2, 4, 12, 24, 36, 52, and 64); (b) proportions of responders over time based on the intra‐individual change of ≥1 or ≥2 grade(s) in WSRS scores compared to preinjection; (c) scores on the Global Aesthetic Improvement Scale (GAIS), as rated by the BLE and subjects; GAIS is a subjective five‐point scale graded from 1 (very much improved; optimal cosmetic results) to 5 (appearance is worse than the original condition)^13^; (d) patient‐reported outcome measures (PROM) assessing subject perception of the aesthetic treatment, using the five questions of the NLF domain of the FACE‐Q questionnaire (adapted to a 100‐unit scale, highest score = 100). FACE‐Q is a validated questionnaire composed of several independently functioning scales that measure outcomes for patients undergoing a multitude of (cosmetic) facial procedures^14^, ^15^; (e) PROM assessing subject overall satisfaction using an ad hoc 5‐grade structured scale (very satisfied, satisfied, neither satisfied nor dissatisfied, dissatisfied, very dissatisfied); (f) number of treatment sessions and total filler volume required to obtain optimal aesthetic results.

### 2.4 Safety assessments

Subjects graded injection site pain using a visual analog scale (VAS) during and at 5, 10, 15, and 30 minutes after each injection session. Safety was evaluated by means of common treatment reactions (CTRs, reported during the follow‐up period on a diary log) and adverse events (AEs). The following events were considered CTRs: bruising, discoloration, firmness, itching, lump/bumps, pain, redness, swelling, and tenderness. Any CTR extending past 14 days was then recorded as an AE.

### 2.5 Statistical analysis

Per‐protocol (PP) population, intention‐to‐treat (ITT) population, and safety (SAFT) population were defined for statistical analysis. Missing data were imputed using last observation carried forward (LOCF) for the WSRS. Unless otherwise stated, all efficacy results are calculated strictly on the PP population and might not represent other calculations requested for FDA labeling approval. Untreated control subjects were not part of the analysis populations. Quantitative variables were described as the mean and standard deviation (SD), whereas categorical variables were described as frequency and percentage.

The primary endpoint was analyzed in a noninferiority statistical model using WSRS scores rated by the BLE at week 24. As a WSRS score change of 1 grade was considered clinically significant, a preinjection and postinjection difference of ≤0.5 grade between the two treatment groups was used as the noninferiority margin. That is, the upper limit of the 95% bilateral confidence interval (CI) had to be ≤0.5 to achieve a noninferiority margin between RHA4 and Lyft. A minimum of 45 subjects per treatment were considered necessary to detect a 0.5 noninferiority (ie, primary endpoint) with a power of 80%. Nevertheless, the recruitment was extended to 120 treated subjects to detect a sufficient rate of AEs and 140 total participants to allocate four untreated subjects at each of the five investigational centers.

Other statistical inference tests were performed using two‐sided tests, with a significance level (α) of 0.05. Mean changes in scores were analyzed using a paired *t* test, whereas responder rates improvement proportions in each treatment group were compared using a paired McNemar's test.

If noninferiority was achieved, WSRS scores, as rated by the BLE at week 24, were to be analyzed in terms of superiority. The upper limit of the 95% bilateral confidence interval had to be <0 to achieve superiority over control treatment. Also, to confirm superiority, ≥50% of the subjects were to demonstrate ≥2‐point WSRS improvement over control treatment. The confirmatory analysis of the primary endpoint was the comparison of the proportion of NLFs on both sides, having a ≥1‐grade improvement on the WSRS over baseline at week 24 (Visit 7; BLE assessments). To achieve superiority, the observed *P*‐value was to be ≤.025 according to the McNemar's test. All statistics were performed using SAS, 9.3 (SAS Institute Inc., Cary, NC, USA).

## 3 RESULTS

### 3.1 Study subjects and treatment

**Figure 2:**
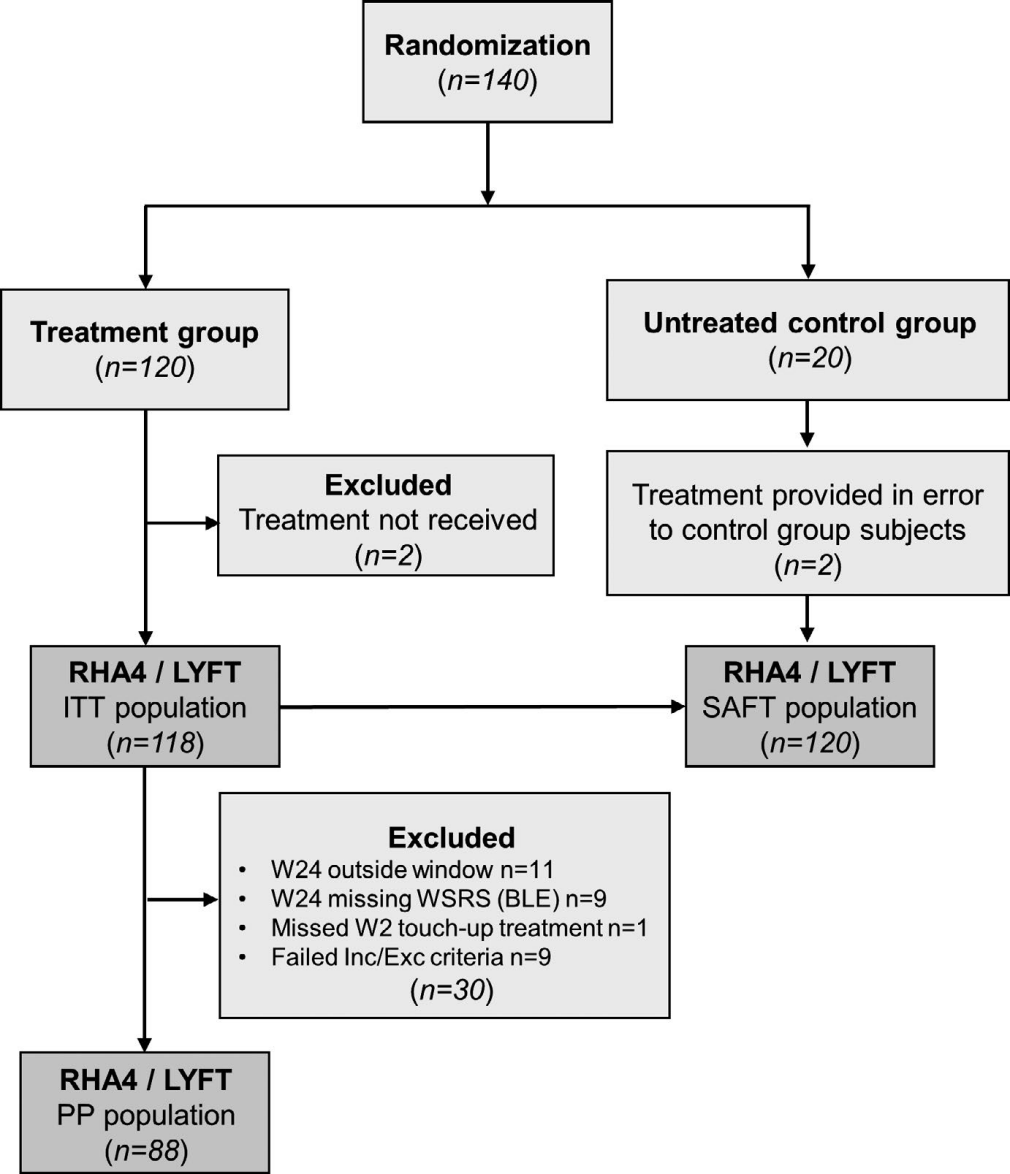
Flowchart of subjects’ inclusion (ConSORT)

Out of 140 randomized subjects who met all selection criteria, 120 were allocated to the active treatment group and 20 to the untreated control group (Figure 2). Two subjects in the active treatment group did not receive treatment, resulting in an ITT population of 118 subjects, out of which 30 were excluded for having one or more protocol violations, resulting in an analysis sample of 88 subjects in the PP population. All subjects who received treatment were considered for the safety analysis: n = 120 for the SAFT population (Figure 2). Subjects in the PP analysis group were mostly Caucasian women and had a mean (±SD) age of 57 (±9) years. All Fitzpatrick skin phototypes were well represented with 61.4% of subjects having skin types I‐to‐III and 38.6% with skin types IV‐VI. The mean initial preinjection WSRS score was 3.49 (±0.50) at each hemiface (Table 1).

**Table 1:** Demographic characteristics of study subjects included in the treatment group

	ITT population (n = 118)	PP population (n = 88)
**Age (years)**		
Mean ± SD	57.4 ± 10.0	57.4 ± 9.3
Median	58.0	57.5
Min‐Max	27.0‐86.0	38.0‐82.0
**Gender [n, (%)]**		
Male	12 (10.2)	7 (8.0)
Female	106 (89.8)	81 (92.0)
**Race [n, (%)]**		
Caucasian	97 (82.2)	74 (84.1)
Black	19 (16.1)	13 (14.8)
American Indian/N. Alaskan	1 (0.9)	1 (1.1)
N. Hawaiian/P. Islander	0 (0.0)	0 (0.0)
Asian	1 (0.9)	0 (0.0)
Other	0 (0.0)	0 (0.0)
**Ethnicity [n, (%)]**		
Hispanic/Latino	30 (25.4)	22 (25.0)
Not Hispanic/Latino	88 (74.6)	66 (75.0)
**Fitzpatrick skin type [n, (%)]**		
I	4 (3.39)	4 (4.6)
II	21 (17.8)	16 (18.2)
III	40 (33.9)	34 (38.6)
IV	31 (26.3)	19 (21.6)
V	14 (11.9)	9 (10.2)
VI	8 (6.8)	6 (6.8)

Abbreviations: ITT, intention to treat; PP, per protocol.

**Figure 3:**
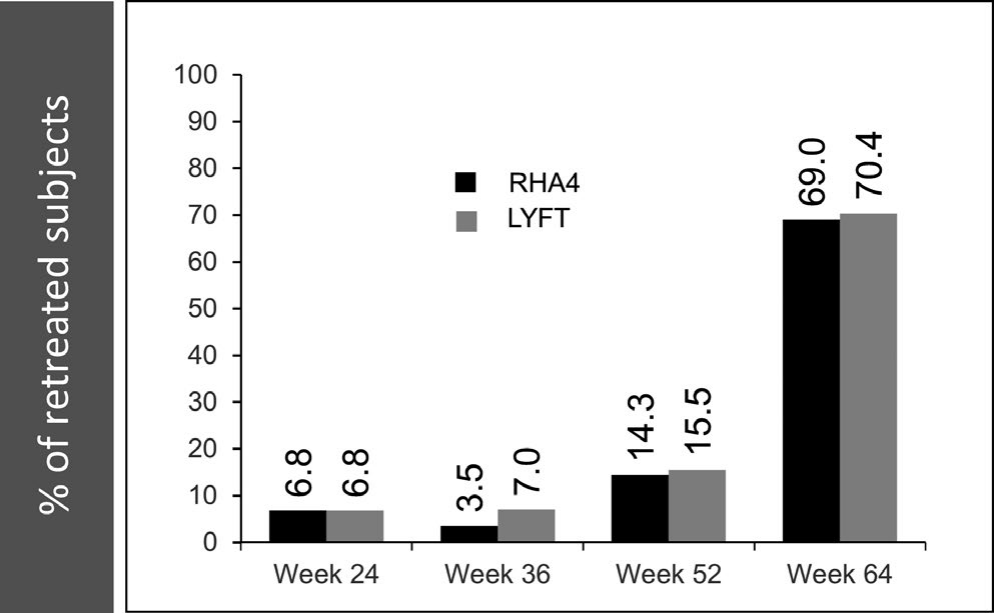
Evolution of the percentage of subjects who underwent retreatment over 15 mo. Data from the PP population

Initially, subjects in the PP population received a mean volume of 1.54 (±0.64) mL of RHA4 and 1.42 (±0.61) mL of Lyft (*P* = .002). Subsequent touch‐up treatments were administered in a significantly (*P* = .012) lower proportion of hemifaces treated with RHA4 (28/88 = 31.8%) than Lyft (38/88 = 43.2%). For those that did receive touch‐up treatments, the mean volume injected did not differ significantly (*P* = .51, NS) between products: 0.79 (±0.31) mL RHA4 vs 0.76 (±0.32) mL for Lyft. Neither did the total volumes (initial + touch‐up) injected 1.79 (±0.87) mL for RHA4 vs 1.75 (±0.90) mL for Lyft, (*P* = .48, NS). Importantly, during subsequent follow‐up, the vast majority of subjects were not retreated before week 52 or mostly week 64 (Figure 3), and were thus followed for at least 1 year (up to 15 months) after their last study treatment. Overall, similar percentages of hemifaces randomized to RHA4 (25.0%) and Lyft (21.6%) did not receive any retreatment during the study. For the 21/88 (23.9%) subjects who did not receive retreatment at week 64, the reasons were that initial results were still optimal according to (a) the treating investigator (33.3%), (b) the subjects themselves (38.1%), or (c) other nonsafety related reasons (28.6%).

### 3.2 Treatment efficacy: wrinkle severity

**Figure 4:**
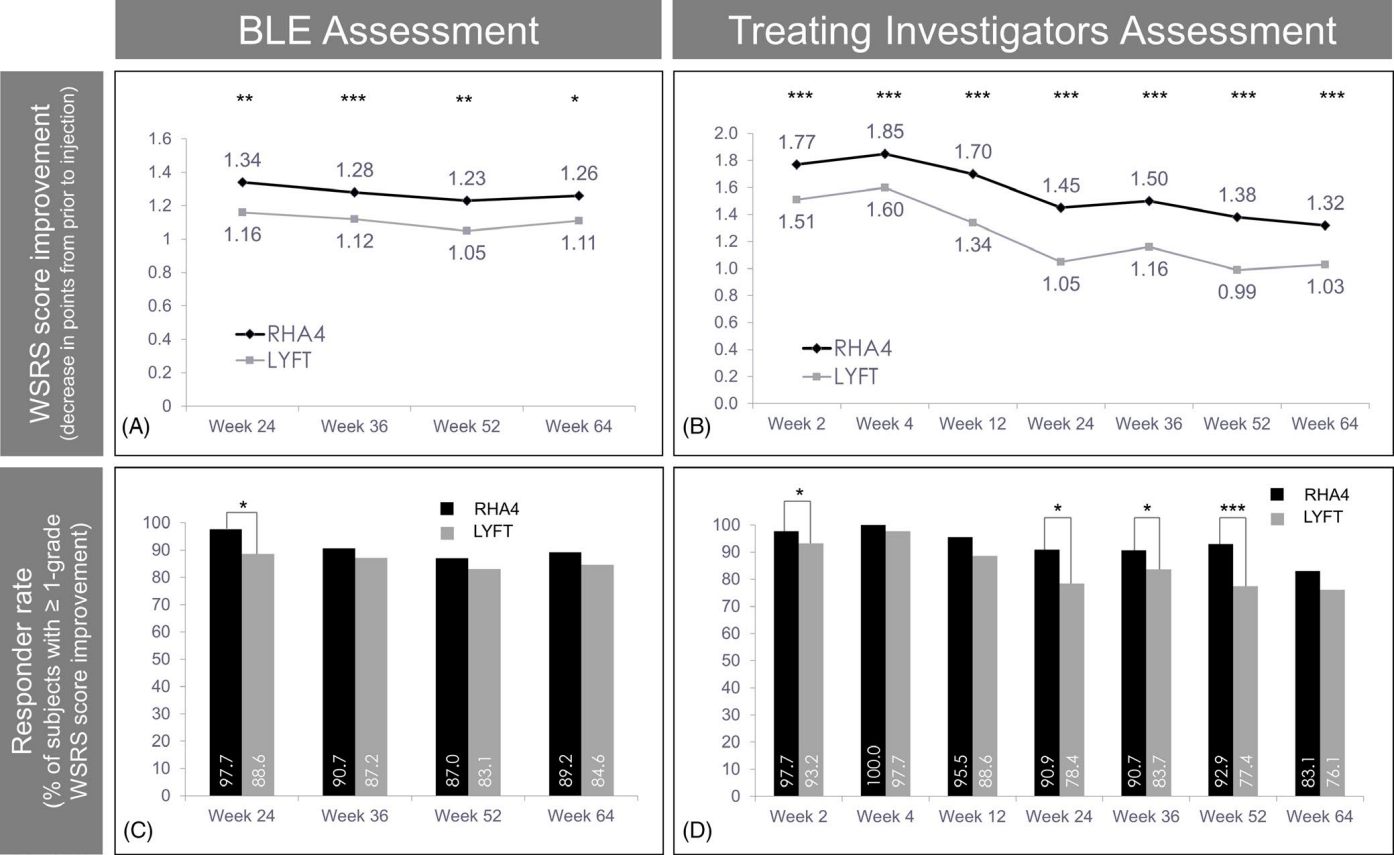
Wrinkle severity. Evolution of WSRS scores (A and B) and 1‐point WSRS responder rates (C and D) according to the BLE (A and C) and treating investigators (B and D) over 15 mo. In this figure and subsequent ones: (*) *P* ≤ .05, (**) *P* ≤ .01, (***) *P* ≤ .001. Data from the PP population

In the PP population, according to BLE ratings, both treatments resulted in a reduction of mean WSRS scores at week 24 post‐treatment. This result was maintained until week 64 (Figure 4A). At week 24, a significantly (*P* = .001) greater mean WSRS score improvement (*ie,* decrease between pre‐ and postinjection) was observed for RHA4 [−1.34 (95% CI −1.46, −1.22)] than for Lyft [−1.16 (95% CI −1.29, −1.03)]. The mean WSRS score difference of −0.18 (95% CI −0.29, −0.07) between RHA4 and Lyft showed that, in terms of WSRS score improvement, RHA4 achieved noninferiority (*ie,* upper limit of the 95% CI ≤ 0.5) and even superiority (*ie,* upper limit of the 95% CI < 0) to Lyft at week 24 (primary endpoint), as well as at subsequent time points (Table 2). The sensitivity analysis, which included all subjects who initiated treatment (ITT population), confirmed both the noninferiority and superiority at week 24, with a mean difference between WSRS scores of −0.22 (95% CI −0.34, −0.11). These results mirrored WSRS assessment by treating investigators, which reported a significantly (*P* < .001) greater reduction of the WSRS score for RHA4 (−1.45 [±0.76]) than for Lyft (−1.05 [±0.76]) at week 24, but also throughout the study from week 2 until week 64 (Figure 4B).

**Table 2:** Changes in mean WSRS scores in the PP population over time, as assessed by the BLE

	RHA4	LYFT	Difference between treatments: mean (95% CI)	*P* value
	Change from pretreatment: mean (95% CI)	Change from pretreatment: mean (95% CI)		
Week 24	−1.34 (−1.46, −1.22)	−1.16 (−1.29, −1.03)	−0.18 (−0.29, −0.07)	.001
Week 36	−1.28 (−1.41, −1.14)	−1.12 (−1.25, −0.99)	−0.16 (−0.25, −0.07)	<.001
Week 52	−1.23 (−1.40, −1.07)	−1.05 (−1.20, −0.90)	−0.18 (−0.30, −0.06)	.004
Week 64	−1.26 (−1.44, −1.08)	−1.11 (−1.28, −0.93)	−0.15 (−0.27, −0.04)	.011

According to BLE ratings, the 1‐point WSRS responder rate (*ie,* the percentage of patients with ≥1 point improvement of the WSRS score) at week 24 was significantly (*P* = .005) greater for RHA4 (97.7%) than Lyft: (88.6%). In subsequent assessments (weeks 36, 52, 64), the RHA4 responder rate remained ≥87% and showed a persistent (though nonsignificant) trend toward higher scores than Lyft (Figure 4C). Analysis of the ITT population confirmed a significantly (*P* = .002) greater WSRS responder rate for RHA4 (97.2%) than Lyft (87.9%) at week 24 (BLE ratings). These results mirrored the 1‐point WSRS responder rate assessment by treating investigators, which showed a lasting trend toward higher scores with RHA4 than Lyft throughout the study, reaching statistical significance at weeks 2, 24, 36, and 52 (Figure 4D). The 2‐point WSRS responder rates at week 24 were 34.1% (95% CI 24.3, 45.0) for RHA4 and 26.1% (95% CI 17.3, 36.6) for Lyft (*P* = .052, NS)(BLE ratings), which represented a 1.31‐fold higher score for RHA4 compared to Lyft. However, the additional superiority criterion of a ≥1.50‐fold greater 2‐point WSRS responder rate for RHA4 was thus not met.

### 3.3 Treatment efficacy: aesthetic improvement

**Figure 5:**
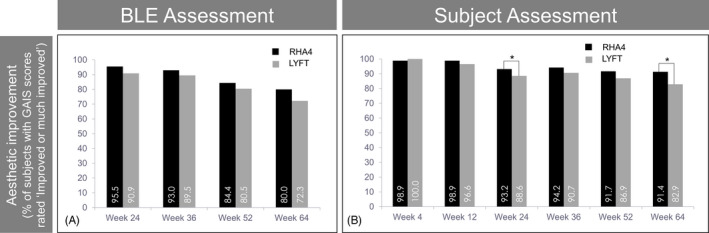
Global Aesthetic Improvement. Evolution of the percentage of subjects rated as “improved” or much improved” on the GAIS, according to the BLE (A) and the subjects themselves (B), over 15 mo. Data from the PP population

In the PP population, for both products, and at all time points assessed, GAIS scores for the vast majority of subjects (>72%) were rated as “improved” or “much improved” from prior to treatment, either by the BLE or the subjects themselves (Figure 5A and B). At all time points, GAIS scores (BLE and subject ratings) showed that the percentages of “improved” or “much improved” subjects with RHA4 were at least equivalent to Lyft. At week 64, that percentage was still 80.0% for RHA4% vs 72.3% for Lyft (*P* = .06, NS) when assessed by the BLE. This difference reached statistical significance (*P* = .014) when assessed by the subjects (91.4% [RHA4] vs 82.9% [Lyft]). Overall, these results support the long‐lasting aesthetic improvement associated with both gels, and the high efficacy of RHA4 throughout the study period.

### 3.4 Subject perception

**Figure 6:**
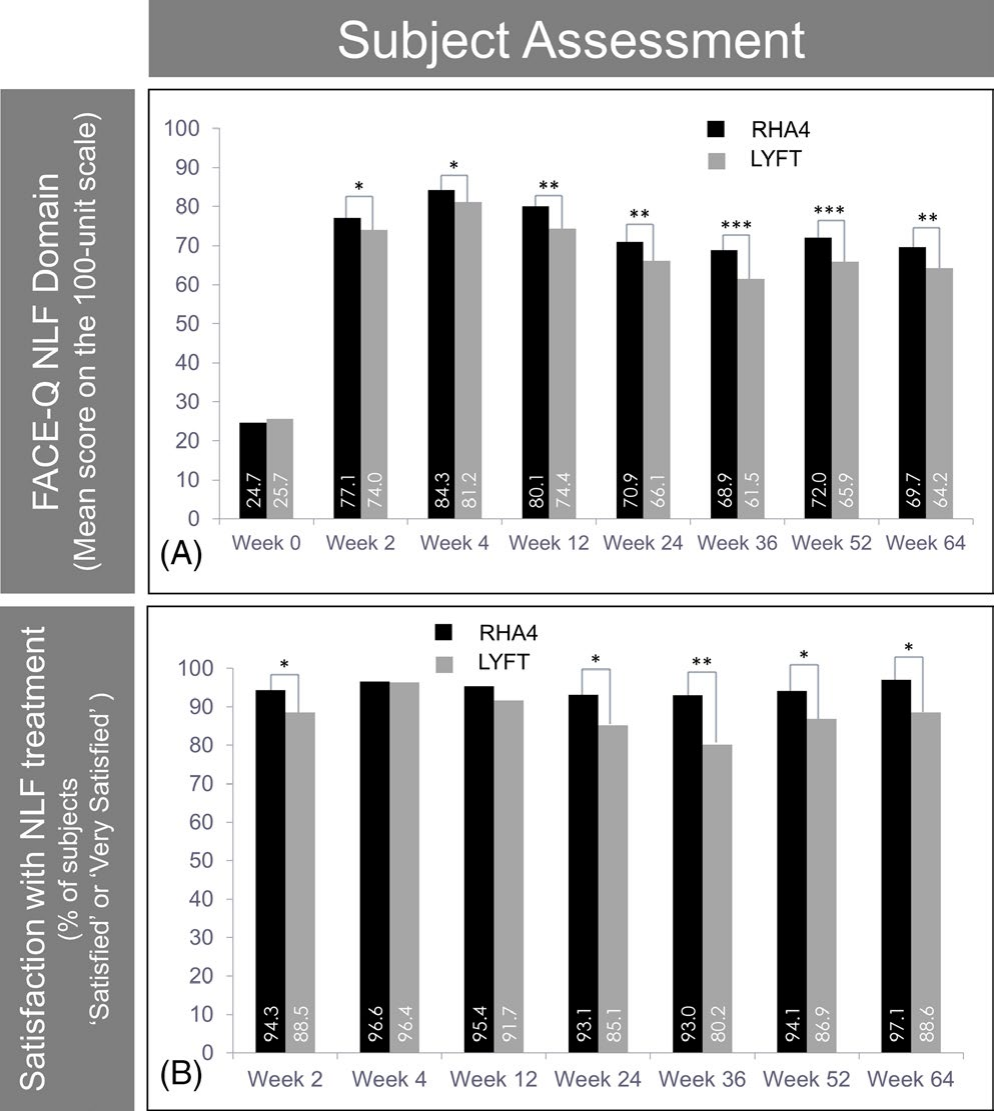
Subjects’ perception and satisfaction with treatment efficacy. Evolution of the mean subject score on the NLF domain of the FACE‐Q questionnaire (100 units) (A) and the percentage of subjects “satisfied” or “very satisfied” on a 5‐point ad hoc satisfaction scale (B) over 15 mo. Data from the PP population

Subjects were asked to assess their NLF and deem how the treatment influenced their quality of life and self‐image using the psychometric FACE‐Q questionnaire (NLF domain). No significant differences were found between hemifaces at the pretreatment visit. Conversely, RHA4 showed consistently higher scores than Lyft after treatment, with significant differences at all assessment points from week 2 onwards (Figure 6A). Likewise, the change in the FACE‐Q scores from pretreatment was significantly higher with RHA4 at all assessment points until week 64.

As a whole, subject satisfaction using a 5‐grade structured scale was also higher with RHA4 than Lyft at all assessment points, except week 4. In addition, a higher percentage of subjects either “satisfied” or “very satisfied” with RHA4 than with Lyft was reported throughout the study, reaching statistical significance at all assessments except weeks 4 and 12 (Figure 6B). Overall, subject satisfaction was high throughout the study for both fillers with scores over 80% until week 64.

### 3.5 Safety

Injection pain was virtually absent 5 minutes postinjection, and there were no clinically meaningful differences between devices regarding pain. Both treatments were safe and well tolerated. The proportions of subjects experiencing at least one CTR (*eg,* bruising, firmness) were comparable between treatment groups and the majority had resolved by Day 14. No deaths or early study withdrawal due to treatment‐related AEs (TRAEs) occurred during the follow‐up period. Three subjects experienced a Serious AE (SAE: arthralgia, diverticulitis, and lung infection), but none was deemed to be related to the study treatment. No vascular occlusion‐related events were reported. TRAEs were based on CTR diary entries and were those commonly expected from the injection of a dermal filler. Overall, the frequencies of TRAEs were consistent with expected incidence rates and similar between treatment groups. Seventy‐one (59%) and 62 (52%) subjects experienced a TRAE related to RHA4 and Lyft, respectively. All TRAEs were mild‐to‐moderate (no severe TRAE), and 334 of 364 events reported (92%) were administration site conditions. Overall, most frequent TRAEs were injection lumps/bumps (n = 55/120, 45.8% of subjects), injection site firmness (n = 49/120, 40.8%), injection site swelling (n = 27/120, 22.5%), and tenderness (n = 21/120, 17.5%). There were no reports of late‐onset TRAEs or granulomas with either filler. The global incidence of TRAEs was higher in subjects with Fitzpatrick skin types I‐III (n = 48, 71.6%) than in those with types IV‐VI (n = 29, 54.7%).

## 4 DISCUSSION

In recent years, the world market for HA‐based dermal fillers has experienced tremendous growth. However, despite the widespread acceptance of their efficacy and safety, many of them lack robust supporting scientific evidence, such as randomized controlled trials, particularly those marketed only in Europe.^2^ RHA4 belongs to a new generation of HA‐based dermal fillers characterized by a crosslinking technology developed to preserve high molecular weight HA chains and decrease their BDDE crosslinking rate, thus conferring mechanical resilience and tissue biointegration to these gels.^10^

The clinical efficacy of RHA4 was assessed by testing its noninferiority to Lyft, a popular and effective dermal filler already commercially available in the US, which has been used in various trials as a reference treatment to investigate the clinical efficacy of other HA‐based fillers.^16^ Like RHA4, Lyft is indicated for deep‐dermal to superficial subcutaneous implantation for the correction of moderate‐to‐severe facial wrinkles and folds such as NLF, with a long history of positive results on the treatment of skin folds, including severe NLF.^5^, ^16^ Our primary endpoint to assess the noninferiority of RHA4 compared to Lyft was the reduction in the WSRS score rated by a BLE.

This study demonstrates the efficacy and safety of RHA4 in the treatment of moderate‐to‐severe NLF. The administration of both fillers resulted in a significant reduction in the WSRS score according to both the BLE and treating investigators. However, the decrease with RHA4 was significantly greater than with Lyft, not only at week 24 (primary endpoint) but also more globally throughout the study from weeks 2 to 64, as rated by the BLE and treating investigators. In terms of WSRS score improvement, RHA4 achieved superiority to Lyft. The mean WSRS score reduction observed with RHA4 (*ie*, 1.34 points) was in the range of that observed in previous trials assessing the efficacy of HA‐based fillers in the correction of severe NLF, at 24 weeks postintervention.^5^, ^17^, ^18^ The 1‐point WSRS responder rate was also significantly higher (*P* = .005) for RHA4 than Lyft at week 24, and a trend for higher rates was maintained throughout the study. Of note, all these results were confirmed in the ITT population (including subjects with protocol deviations) and when considering the assessment by treating investigators. Statistical superiority of RHA4 over Lyft was achieved in terms of WSRS score reduction at week 24 (and all subsequent time points), as well as one‐point WSRS responder rates. However, it was not formally confirmed by the analysis of the two‐point WSRS responder rate.

The GAIS is more subjective than the WSRS, but it is one of the most informative tools for the global appraisal of aesthetic outcomes associated with changes in folds and wrinkles severity after treatments.^19^ In our study, both the BLE and subjects reported GAIS scores for RHA4 that were at least equivalent to Lyft until study completion. At week 64, global aesthetic improvement was still reported by 80.0% (RHA4) vs 72.3% (Lyft) of the subjects (according to the BLE), and by 91.4% (RHA4) vs 83.9% (Lyft) of the subjects themselves, this latter difference being statistically significant.

In addition to the specific assessment of NLF severity and overall aesthetic improvement, subjects’ subjective perception regarding the treatment performed with dermal fillers is also vital; an improved self‐image is perhaps the most relevant goal of aesthetic interventions. In our study, this assessment was carried out using both a structured ad hoc five‐point satisfaction scale and the NLF domain of the FACE‐Q, a widely used questionnaire originally developed as a PROM of the effectiveness of facial aesthetic procedures.^14^ RHA4 scores were higher than Lyft in both assessments and at all time points until week 64, the difference being statistically significant for all FACE‐Q results, and for satisfaction results at most time points from week 2 onwards.

Importantly, all assessments demonstrated that RHA4 provides a long‐lasting correction of moderate‐to‐severe NLF and long‐term subject satisfaction with the treatment, up to 15 months. While this time period exceeds that of several previous trials assessing the effectiveness of dermal fillers for correcting NLF,^5^, ^17^, ^18^, ^20^ longer follow‐up periods have also been reported.^21^ Long‐lasting patient satisfaction with RHA4 was also confirmed by the low rates of retreatments observed until study completion at week 64. With retreatment being offered unconditionally (and without cost to the subject) at the final step of the study, it is noteworthy that 25% of RHA4 hemifaces never underwent any retreatment with the primary reason cited being that correction was still optimal.

For both treatments, CTRs were comparable and the majority did not extend beyond 14 days (last day of reporting on the subject diary log). All TRAEs were mild‐to‐moderate in severity and were consistent with those experienced with other injectable HA fillers.^22^ No late‐onset TRAEs or granulomas were reported. Importantly, darker Fitzpatrick skin phototypes (IV‐VI) showed an even lower incidence of TRAEs than lighter types (I‐III), confirming the consistent safety of all products tested in all skin types.

Overall, our results are strengthened by the robustness of the noninferiority design, which may challenge results interpretation, but allows to draw robust conclusions and offers the ability to identify innovative treatments.^23^ While aesthetic dermatology procedures represent an increasing demand among males, they may have a higher threshold for seeking cosmetic interventions. Similar to previous trials assessing the efficacy of aesthetical interventions, men were underrepresented in our study sample (8% of the PP population). Future gender‐specific investigations may provide complementary information on the efficacy of RHA4 for NLF in each gender.

In summary, our results consistently demonstrate a high efficacy and long‐lasting effect of RHA4 in the correction of NLF that can be safely and reliably used in patients of various Fitzpatrick skin types.

## CONFLICT OF INTEREST

JKJ received research funding from TEOXANE SA and served on the advisory board for this study. She also has received research funding from Galderma SA in the past. SCT received research support from TEOXANE SA for this study, as well as from Medicis Pharmaceutical Corp. (Perlane Trial) in the past. SEC received no grant, research support, honoraria or consulting fee for this report except payment to Aesthetic Solutions for the conduct of the clinical study. SHW received research support from TEOXANE SA and was an advisory board member for this study. SS received fees/ honoraria as a consultant for TEOXANE SA and Galderma SA. BMK received research support from TEOXANE SA for this study.

## References

[1] Kablik J , Monheit GD , Yu LP , Chang G , Gershkovich J . Comparative physical properties of hyaluronic acid dermal fillers. Dermatologic Surg. 2009;35:302‐312. 10.1111/j.1524-4725.2008.01046.x 19207319

[2] Breithaupt AD , Custis T , Beddingfield F . Next‐generation dermal fillers and volumizers. Cosmet Dermatology. 2012;25:184.

[3] Salwowska NM , Bebenek KA , Żądło DA , Wcisło‐Dziadecka DL . Physiochemical properties and application of hyaluronic acid: a systematic review. J Cosmet Dermatol. 2016;15:520‐526. 27324942 10.1111/jocd.12237

[4] Wang C , Tammi M , Tammi R . Distribution of hyaluronan and its CD44 receptor in the epithelia of human skin appendages. Histochem Cell Biol. 1992;98:105‐112. 10.1007/BF00717001 1429018

[5] Buntrock H , Reuther T , Prager W , Kerscher M . Efficacy, safety, and patient satisfaction of a monophasic cohesive polydensified matrix versus a biphasic nonanimal stabilized hyaluronic acid filler after single injection in nasolabial folds. Dermatologic Surg. 2013;39:1097‐1105. 10.1111/dsu.12177 23506356

[6] Pierre S , Liew S , Bernardin A . Basics of dermal filler rheology. Dermatologic Surg. 2015;41:S120‐S126. 10.1097/DSS.0000000000000334 25828036

[7] Edsman K , Nord LI , Öhrlund Å , Lärkner H , Kenne AH . Gel properties of hyaluronic acid dermal fillers. Dermatologic Surg. 2012;38:1170‐1179. 10.1111/j.1524-4725.2012.02472.x 22759254

[8] Michaud T , Gassia V , Belhaouari L . Facial dynamics and emotional expressions in facial aging treatments. J Cosmet Dermatol. 2015;14:9‐21. 25620090 10.1111/jocd.12128

[9] Sundaram H , Mackiewicz N , Burton E , Peno‐Mazzarino L , Lati E , Meunier S . Pilot comparative study of the topical action of a novel, crosslinked resilient hyaluronic acid on skin hydration and barrier function in a dynamic, three‐dimensional human explant model. J drugs dermatology. 2016;15:434‐441. 27050698

[10] Micheels P , Sarazin D , Besse S , Elias B . Comparison of two swiss‐designed hyaluronic acid gels: six‐month clinical follow‐up. J Drugs Dermatol. 2017;16:154‐161. 28300858

[11] Bourdon F , Meunier S 2016 Process for evaluating the mechanical performance of a filler gel. Pat WO 2016/150974 A1 2016;27.

[12] Day DJ , Littler CM , Swift RW , Gottlieb S . The wrinkle severity rating scale: a validation study. Am J Clin Dermatol. 2004;5:49‐52. 14979743 10.2165/00128071-200405010-00007

[13] Narins RS , Brandt F , Leyden J , Lorenc ZP , Rubin M , Smith S . A randomized double‐Blind, multicenter comparison of the efficacy and tolerability of restylane versus zyplast for the correction of nasolabial fol. Dermatologic Surg. 2003;29:588‐595. 10.1046/j.1524-4725.2003.29150.x 12786700

[14] Klassen AF , Cano SJ , Scott A , Snell L , Pusic AL . Measuring patient‐reported outcomes in facial aesthetic patients: development of the FACE‐Q. Facial Plast Surg. 2010;26:303‐309. 20665408 10.1055/s-0030-1262313

[15] Kappos EA , Temp M , Schaefer DJ , Haug M , Kalbermatten DF , Toth BA . Validating facial aesthetic surgery results with the FACE‐Q. Plast Reconstr Surg. 2017;139:839‐845. 28002252 10.1097/PRS.0000000000003164

[16] Brandt FS , Cazzaniga A . Hyaluronic acid fillers: restylane and perlane. Facial Plast Surg Clin North Am. 2007;15(1):63‐76. 17317557 10.1016/j.fsc.2006.11.002

[17] Park KY , Ko EJ , Kim BJ , et al. A multicenter, randomized, double‐blind clinical study to evaluate the efficacy and safety of PP‐501‐B in correction of nasolabial folds. Dermatologic Surg. 2015;41:113‐120. 10.1097/DSS.0000000000000203 25521103

[18] Rzany B , Bayerl C , Bodokh I , et al. Efficacy and safety of a new hyaluronic acid dermal filler in the treatment of moderate nasolabial folds: 6‐month interim results of a randomized, evaluator‐blinded, intra‐individual comparison study. J Cosmet Laser Ther. 2011;13:107‐112. 21609212 10.3109/14764172.2011.571699

[19] Carruthers A , Carruthers J . A validated facial grading scale: The future of facial ageing measurement tools? J Cosmet Laser Ther. 2010;12:235‐241. 20825260 10.3109/14764172.2010.514920

[20] Carruthers A , Carey W , De Lorenzi C , Remington K , Schachter D , Sapra S . Randomized, double‐blind comparison of the efficacy of two hyaluronic acid derivatives, restylane perlane and hylaform, in the treatment of nasolabial folds. Dermatol Surg. 2005;31:1591–1598; discussion 1598. 16416643 10.2310/6350.2005.31246

[21] Noh TK , Moon HR , Yu JS , et al. Effects of highly concentrated hyaluronic acid filler on nasolabial fold correction: a 24‐month extension study. J Dermatolog Treat. 2016;27:510‐514. 27121901 10.3109/09546634.2016.1170759

[22] Philipp‐Dormston WG , Bergfeld D , Sommer BM , et al. Consensus statement on prevention and management of adverse effects following rejuvenation procedures with hyaluronic acid‐based fillers. J Eur Acad Dermatol Venereol. 2017;31(7):1088‐1095. 28449190 10.1111/jdv.14295

[23] Mauri L , D'Agostino RB . Challenges in the design and interpretation of noninferiority trials. Drazen JM, Harrington DP, McMurray JJV, Ware JH, Woodcock J, editors. N Engl J Med. 2017;377:1357‐1367. 28976859 10.1056/NEJMra1510063

